# Prevalence and Correlates of Hypertension among Japanese Adults, 1975 to 2010

**DOI:** 10.3390/ijerph15081645

**Published:** 2018-08-03

**Authors:** Katharina Otani, Rei Haruyama, Stuart Gilmour

**Affiliations:** 1 Advanced Therapies Innovation Department, Siemens Healthcare K.K., 1-11-1 Osaki, Shinagawa-ku, Tokyo 141-8644, Japan; 2 Department of Global Health Policy, Graduate School of Medicine, The University of Tokyo, 1-3-7 Hongo Bunkyo-ku, Tokyo 113-0033, Japan; rharuyama.obgyn@gmail.com (R.H.); sgilmour@m.u-tokyo.ac.jp (S.G.); 3 Bureau of International Health Cooperation, National Center for Global Health and Medicine, 1-21-1 Toyama, Shinjuku-ku, Tokyo 162-8655, Japan

**Keywords:** Japan epidemiology, hypertension epidemiology, health surveys, public health

## Abstract

We investigated the prevalence and factors associated with hypertension, its treatment, and control using individual-level data from 300,249 respondents aged 20 years and older from the Japanese National Health and Nutrition Survey for the period of 1975–2010. We applied multivariate random effects logistic regression to assess associations between the risk factors and the prevalence of hypertension, the proportion of uncontrolled hypertension, and the proportions of respondents seeking treatment and controlling hypertension. The trends in the effect of the birth cohort on uncontrolled hypertension were also examined. Having hypertension was associated with being male, older, obese, drinking alcohol, and working in the primary industry and a higher proportion of middle-aged men than women were found being obese and drinking alcohol. Seeking treatment was associated with being older, obese, drinking alcohol, working in a primary industry and exercising. Controlling hypertension was associated with being younger, underweight and exercising. The proportion of individuals with uncontrolled hypertension declined for cohorts born in later years with a steeper decline for women than men. Raising awareness in the hypertensive population, especially among men, could help further reduce the prevalence of hypertension in Japan.

## 1. Introduction

Hypertension remains a leading global risk factor for death and disability. In 2011 the World Health Organization (WHO, Geneva, Switzerland) targeted a 25% global reduction in prevalence of hypertension by 2025 [1], but progress varies widely between countries. Over the past 50 years, the mean blood pressure of the Japanese population has seen a steady decline, but hypertension and smoking still remain the two biggest risk factors for non-communicable diseases, in particular, cardiovascular disease [2,3,4]. Understanding the trends in hypertension prevalence and management is essential to preparing strategies to target this risk factor in Japan. We used individual-level data from the Japanese National Health and Nutrition Survey (NHNS) from 1986 to 2010, and, where available, from 1975 to 2010, to explore the trends and risk factors for hypertension in Japan. We thus expand on previous investigations of blood pressure in the Japanese population [5,6,7] and aimed to:

- Analyze trends in and risk factors for uncontrolled hypertension over 35 years and for hypertension over 25 years
- Identify factors associated with seeking treatment and controlling hypertension
- Investigate changing patterns in the proportion of individuals with uncontrolled hypertension by the birth cohort over 35 years.

Japan has made progress in reducing hypertension and improving management of this condition, but further work is needed. The results of this study may be used to plan interventions in specific population groups at risk of hypertension, and to raise awareness of the need for the identification, treatment, and control of hypertension.

## 2. Method

### 2.1. Data Sources

This study used data from the NHNS between 1975 and 2010. The NHNS is a repeated cross-sectional survey conducted annually on a random sample of Japanese citizens in all 47 Japanese prefectures. The sample size was about 28,000 persons in 1975, 20,000 persons between 1976 and 1986, and gradually decreased to about 10,000 or less after 2000 [8,9]. In addition to answering a questionnaire, the respondents were invited to undergo a physical examination that included blood pressure measurements. The response rate to the physical examination was reported as around 50 to 60% [9,10]. Stratified randomization by prefecture and household in a two-stage cluster sampling design is used for participant selection [8,9,11]. Here, we restricted the analysis to respondents who were at least 20 years old, not pregnant, and whose systolic and diastolic blood pressure data were available. Fifteen-year age brackets were chosen to ensure sufficient numbers of respondents in each age category.

### 2.2. Blood Pressure Measurement and Hypertension

Data on blood pressure values were available from 1975 to 2010 and data on hypertension medication were available from 1986 to 2010. Until 1999, the survey included one measurement of blood pressure in the sitting position, and from 2000 it included two measurements of blood pressure measured in the sitting position with a Riva-Rocci mercury sphygmomanometer after resting [5,11,12]. The two measurements were averaged for analysis, consistent with the Japanese Ministry of Health, Labour and Welfare guidelines [13].

Hypertension was defined as a systolic blood pressure of at least 140 mmHg or a diastolic blood pressure of at least 90 mmHg or the use of medication for hypertension, in line with the current World Health Organization (WHO, Geneva, Switzerland) guidelines [14,15]. Uncontrolled hypertension was defined in the same way except that respondents who successfully controlled their blood pressure to the normal range by medication were counted as controlled. Values of 50 to less than 300 mmHg for systolic blood pressure and 40 to less than 140 mmHg for diastolic blood pressure were considered valid. Values outside these extremes were omitted. Medicated hypertension was defined as taking medication for hypertension regardless of successful control or not. Controlled hypertension was defined as medicated hypertension with a measured blood pressure in the normal range. Since information on medication was only available after 1986, the prevalence of hypertension and the proportions of medicated hypertension and controlled hypertension were analyzed starting from the survey year 1986.

### 2.3. Correlates of Hypertension

In its guidelines, the Japanese Society of Hypertension defines the male sex, advanced age, high body mass index (BMI), not exercising, high salt intake, low vegetable and fruit intake, regularly drinking alcohol and smoking as risk factors for hypertension [16]. Job strain, defined as a low control over work-related decisions and having a high workload, has also been reported as a risk factor [17,18] as has socioeconomic status [19]. Risk factors available in the NHNS were sex, age, BMI (1975–2010), salt intake (2000–2010), exercising (1986–2010, 2008 and 2009 missing), drinking alcohol (1986–2010), current smoking status (1986–2010), and occupation (1975–2010). The BMI was calculated as weight in kilograms divided by the square of height in meters, omitting values of weight below 20 kg and of height below one meter. The BMI values of 10 to less than 70 kg/m^2^ were retained as these have been defined elsewhere as plausible limits [20,21]. The BMI was classified following WHO guidelines: underweight was defined as BMI less than 18.5 kg/m^2^; normal as the BMI values from 18.5 to 25 kg/m^2^; overweight as the BMI values over 25 kg/m^2^ and obese as 30 kg/m^2^ or more [22]. For the risk factor analysis, overweight and obese subjects were collapsed into one category of obese defined as 25 kg/m^2^ BMI or more because Asian populations have been found to be at risk for metabolic disease at lower BMI values than Western populations [23]. In the NHNS, the dietary sodium intake is estimated retrospectively from self-reported food intake [9]. We converted sodium intake to salt intake by multiplication with a factor of 2.54 [24]. Drinking alcohol was defined as regularly drinking more than one glass of Japanese sake (180 mL), one bottle of beer (500 mL), or one double whiskey (60 mL) at least four times a week [5,11]. Exercising was defined as physical activity for more than 30 min at least three times a week for over one year.

Occupations were classified into primary, secondary, and tertiary industries, in line with the Japanese Ministry of Internal Affairs and Communication [25]. This classification would to some extent reflect socioeconomic status, as the primary industries include workers in farms and fisheries, the secondary industries include a large proportion of blue collar workers and the tertiary industries include the largest proportion of white-collar workers and persons with high education levels among the three industries. 

### 2.4. Statistical Analysis

All statistical analyses were performed with a commercially available software package (Stata14, StataCorp LP, College Station, TX, USA). We applied multivariate random effects logistic regression to assess the association of hypertension, uncontrolled hypertension, treated hypertension, and controlled hypertension with the covariates, including a random intercept term for prefecture to account for clustering within and unmeasured variations between prefectures. Graphs of the proportion of individuals with uncontrolled hypertension by birth year for birth cohorts were smoothed using a kernel-weighted local polynomial regression smoother.

## 3. Results

### 3.1. Demographic Characteristics of the Sample

There were 554,544 observations available after the exclusion of pregnant women. After excluding 5 respondents because of anomalies between gestational age and pregnancy variables, 149,846 respondents because they were less than 20 years old, and 104,210 missing and 54 unrealistic blood pressure measurements, the final sample consisted of 300,429 respondents sampled between 1975 and 2010. The mean age was 49.9 ± 16.2 years. Among the respondents, 124,871 were men and 175,558 women with mean ages of 49.6 ± 16.2 years and 49.4 ± 16.1 years, respectively. The mean BMI was 22.9 ± 3.3 kg/m^2^ for all respondents, 23.2 ± 3.1 kg/m^2^ for men and 22.7 ± 3.5 kg/m^2^ for women. The mean salt intake was 11.7 ± 4.8 g/day for all respondents, 12.7 ± 4.8 g/day for men and 11.0 ± 4.8 g/day for women. Table 1 gives an overview of the years of data availability, and the frequencies and proportions of characteristics of the sample.

### 3.2. Prevalence of Hypertension and Proportions of Uncontrolled Hypertension, Treatment, and Control

**Figure 1:**
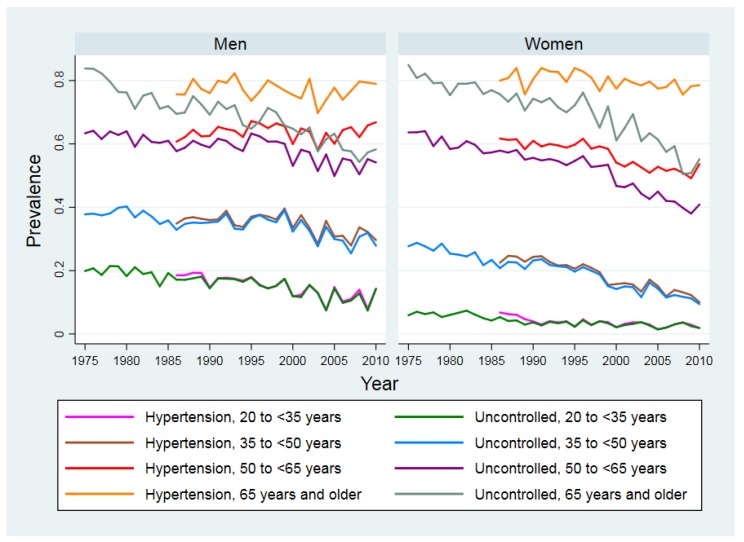
The prevalence of hypertension (1986–2010) and the proportion of individuals with uncontrolled hypertension (1975–2010) in Japan by sex and age.

Figure 1 shows the trends in the prevalence of hypertension (1986–2010) and the proportion of individuals with uncontrolled hypertension (1975–2010) by sex and age. Between 1986 and 2010, the prevalence of hypertension has not changed much in men and women of 65 years and older but appears to increase in middle-aged men of 50 to less than 65 years old. In contrast, for men younger than 50 and women younger than 65 years, the prevalence of hypertension and the proportion of individuals with uncontrolled hypertension both showed decreasing trends. 

Table 2 shows the prevalence of hypertension (1986–2010), the proportion of individuals with uncontrolled hypertension (1975–2010), the proportion of hypertensive respondents receiving medication (1986–2010), and the proportion of individuals with controlled hypertension (1986—2010) among medicated respondents stratified by sex and age. The prevalence of hypertension increased with age and was higher among men than women, except for the age group of 65 and older. The proportion of hypertensive respondents seeking treatment increased with age, as did the proportion of respondents who controlled their hypertension with higher proportions of women than men for both. Men of 65 years and older were, however, equally or more successful in controlling their hypertension than women.

### 3.3. Risk Factors Associated with Hypertension

The means and proportions of the risk factors of age, salt intake, BMI, smoking, drinking alcohol, exercising and occupation are shown in Table 3 for one year in the beginning, in the middle and at the end of the period of analysis. Differences between men and women were seen in trends of BMI, smoking, and drinking. The proportion of women who smoke and regularly drink alcohol was still much lower in 2010 than that of men although the difference has narrowed in more recent years compared to the year 1975. Table 4 shows the odds ratios of having hypertension for each factor adjusted for all other factors. Older age was the strongest predictor (OR 42.85, 95% CI 39.93–46.00, for at least 65 compared to 20 to less than 35 years old). The strongest modifiable risk factor was BMI (OR 2.55, 95% CI 2.48–2.62 for obese compared to normal weight people). Salt intake and exercise showed no effect. Occupation had a weak association with hypertension.

### 3.4. Factors Associated with the Treatment and Control of Hypertension

Table 4 also shows the association of each risk factor with the treatment and control of hypertension adjusted for all other covariates. The odds of taking medication (OR 1.02, 95% CI 1.02–1.02) and the odds of controlling hypertension (OR 1.05, 95% CI 1.04–1.05) increased year by year. The odds of taking medication increased with age. The odds of controlling hypertension among those who sought treatment was lower for those aged 50 and older than for those younger than 50 years. Obese people had higher odds of seeking treatment than normal weight people (OR 2.07, 95% CI 1.99–2.14), but lower odds of controlling their hypertension (OR 0.75, 95% CI 0.70–0.81). In contrast, underweight people had lower odds of seeking treatment than people of normal weight (OR 0.58, 95% CI 0.53–0.64), but higher odds of controlling their hypertension (OR 1.32, 95% CI 1.10–1.59).

### 3.5. Effect of Birth Cohort on Uncontrolled Hypertension

**Figure 2:**
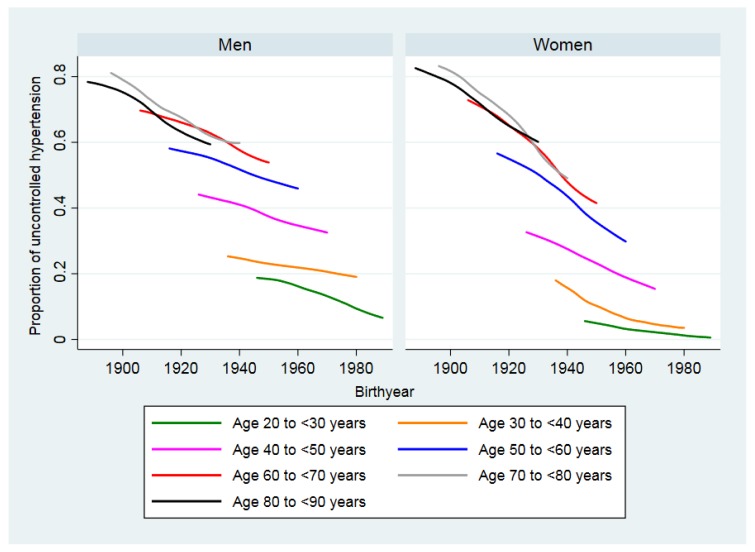
Uncontrolled hypertension in Japan by birth year (1886 to 1989) and sex for birth cohorts.

Figure 2 shows that the proportion of individuals with uncontrolled hypertension by birth year for birth cohorts declined for cohorts born in later years in both men and women of all age groups. The decline appears to be steeper for women than men for all age groups.

## 4. Discussion

This study investigated the prevalence of hypertension and risk factors, the proportions of treatment and control among Japanese adults from 1986 to 2010, the proportion of individuals with uncontrolled hypertension, and the influence of birth cohorts from 1975 to 2010 using a large annual cross-sectional survey. Our study expanded on previous studies based on NHNS data on blood pressure levels [5,7] and on the prevalence of hypertension in Japan [7].

In the time period preceding our data, an analysis of NHNS data collected between 1956 and 1980 showed that the proportion of individuals with uncontrolled hypertension defined as systolic blood pressure over 180 mmHg declined in the age groups over 50 but did not markedly change in the younger population [7]. The proportion of individuals with uncontrolled hypertension, defined as diastolic blood pressure over 100 mmHg, also declined for age groups over 50 but did not markedly change in the younger population [7]. In our analysis of NHNS data between 1975 and 2010, we found that the proportion of individuals with uncontrolled hypertension declined for men and women of all ages with steeper declines in women and in the older population.

The risk factors for having hypertension identified in our study were consistent with those previously reported as being male, of older age, obese, drinking alcohol, and working in the primary industry. These risk factors, except occupation-related factors, were also investigated in a previous analysis of blood pressure levels based on NHNS data between 1986 and 2002 [5] and correlated with having a systolic blood pressure of at least 140 mmHg. Drinking alcohol has consistently been reported as a risk factor in other reports [5,7,26]. In contrast to other investigators [27,28], we did not find evidence of an effect of salt intake on the prevalence of hypertension. One possible reason is that salt intake was estimated from self-reported food intake [8], which may not be precise enough for a quantitative analysis.

In our analysis, smoking appears to have a blood pressure lowering effect, which has also been reported elsewhere [29]. This effect has been a subject of controversy because smoking is a known risk factor for atherosclerosis, which may lead to more severe forms of hypertension [29]. There is agreement that smoking is overall harmful regardless of its potential impact on blood pressure [29].

Our results show that primary industry workers are at increased risk of having hypertension. Although specific information on income or socioeconomic status was not collected in the NHNS, the classification of occupations into industries could serve as a proxy indicator of socioeconomic status in Japan, because more people of lower education and lower socioeconomic status work in the primary and secondary industries. Similar results have been found in Western Europe where the odds of hypertension was inversely associated with the education level [30] and for the US where people of lower socioeconomic status had a higher incidence of hypertension [31].

Our analysis revealed that the prevalence of hypertension declined for men and women except for men between 50 and less than 65. The steepest declines were seen in women of 35 and older. Additionally, the prevalence of hypertension was consistently lower in women than in men of the same age except for women in the age group of 65 and older. Possible explanations for the lower prevalence of hypertension in Japanese women are that they consume less alcohol than men and have a lower BMI than men of the same age [32]. Japan has been successful in managing obesity in women, but obesity prevalence in Japanese men is still increasing [5,32]. We found that the decline in the proportion of individuals with uncontrolled hypertension seemed to have resulted from successful control by medication. A previous report also concluded that control of hypertension by medication was the main reason for a decline of systolic blood pressure in all people, except in young women [5]. Furthermore, we found that birth cohorts of women born between 1920 and 1960 experienced a steeper decrease in the proportion of individuals with uncontrolled hypertension than men born in the same years.

Our data suggest that the proportion of hypertensive respondents who sought treatment and of those who successfully controlled their hypertension has been increasing with time. This confirmed a result from the survey “National Integrated Project for Prospective Observation of Non-communicable Disease and its Trends in the Aged” (NIPPON DATA) 1980 to 2010, a large survey that is undertaken every 10 years [6]. We found that the adjusted odds of seeking treatment were higher for older and obese respondents, alcohol consumers, workers in the primary industry and individuals who exercised. The adjusted odds of controlling hypertension were higher for underweight and younger respondents and individuals who exercised, suggesting that respondents who are more health conscious are also more likely to control their blood pressure. 

In Japan, every employee is required to undergo a yearly health examination that includes blood pressure measurements [33] and every resident 40 years and older regardless of employment can receive an optional inexpensive yearly health examination that includes a blood pressure measurement [34]. Health examinations in middle age have been shown to be associated with a lower utilization of hospital beds by the elderly [34]. Although the early detection of hypertension was not specifically investigated, it may be one of the factors contributing to the lower utilization of hospital beds. 

The global targets for non-communicable diseases for 2025 set by the WHO in 2011 include a reduction in the prevalence of age-standardized hypertension by 25% [1]. In 2009 it was 36.8% (95% CI 35.4–38.3%) in Japan [35] and would thus need to decrease to the level of about 28% to meet the WHO target. The Japanese Society of Hypertension recommends the following public healthcare measures to lower the systolic blood pressure of the Japanese: improved nutrition, more exercise, lower alcohol consumption for men, and seeking treatment [36]. Our results provide further evidence for these measures, in particular, that efforts should be made to ensure that men achieve the same decrease in the prevalence of hypertension as women by addressing obesity and drinking, especially targeting middle-aged men. The prevalence of hypertension among Japanese people of 50 years and older remains high, so raising awareness for seeking treatment and controlling blood pressure should be a priority, in particular, among men. 

There were limitations in this analysis. The blood pressure measurements were changed from one to two measurements in the year 2000. We used the averaged blood pressure values which may have resulted in lower blood pressure than using only the first measurement. The NHNS contained consistent information on hypertension risk factors only for sex, age, BMI and occupation over the whole time period of 1975 to 2010. Data on hypertension medication, smoking, drinking alcohol and exercising started being collected from 1986, data on exercising were missing for the years of 2008 and 2009 and data on individual level salt intake were recorded from 2000. The participation rate of the physical NHNS examination was about 50 to 60% [9,10], which may have introduced selection bias. Randomization schemes and probability weights were not available, thus, we could not carry out a rigorous survey analysis [9,10,37]. We could not quantitatively compare our results with those of previous reports because of differences in definitions and statistical methods.

## 5. Conclusions

The prevalence of hypertension has been declining in Japan in adults except recently in middle-aged men. The proportion of individuals with uncontrolled hypertension has been declining in all adults. Japanese women consume less alcohol than men and have a lower BMI than men which may partly explain the lower prevalence of hypertension in women than in men except in the oldest age group. The proportion of individuals with uncontrolled hypertension declined for cohorts born in later years in both men and women of all age groups with a steeper decline for women than men. Raising awareness for seeking treatment and controlling hypertension among the Japanese hypertensive population, in particular, those over 50 years old and lifestyle changes among men could bring Japan nearer the goal of reducing the burden of hypertension, with considerable positive health impacts in Japan’s aging population.

## Figures and Tables

**Table 1:** The characteristics of the sample.

		Frequency	Proportion in %	Years of Available Data
Sex	Male	124,871	41.56	1975 to 2010
	Female	175,558	58.44	
Age (years)	20 to 35	63,208	21.04	1975 to 2010
	35 to 50	91,366	30.41	
	50 to 65	125,305	41.71	
	At least 65	20,550	6.84	
BMI	Underweight	22,317	7.44	1975 to 2010
	Normal	211,419	70.50	
	Overweight	58,842	19.62	
	Obese	7296	2.43	
Smoking	Non smoker	123,042	73.82	1986 to 2010
	Current smoker	43,640	26.18	
Drinking alcohol	Less than 4 times a week	125,411	75.34	1986 to 2010
	At least 4 times a week	41,040	24.66	
Exercise	Less than 3 times a week	119,814	76.18	1986 to 2010 (2008, 2009 missing)
	At least 3 times a week	37,462	23.82	
Occupation	Primary industry	25,782	9.73	1975 to 2010
	Secondary industry	52,622	19.87	
	Tertiary industry	186,485	70.40	

**Table 2:** The prevalence of hypertension (1986–2010) and the proportions of individuals with uncontrolled hypertension (1975–2010), medicated hypertension (1986–2020) and controlled hypertension (1986–2010).

	Age (years)	Hypertension (1986–2010, *n* = 167,506)		Uncontrolled Hypertension (1975–2010, *n* = 300,429)		Medicated Hypertension (1986–2010, *n* = 73,758)		Controlled Hypertension (1986–2010, *n* = 28,519)	
		Frequency in Each Age Group (Age Group Total)	Proportion (%) in Each Age Group (95% CI)	Frequency in Each Age Group (Age Group Total)	Proportion (%) in Each Age Group (95% CI)	Frequency in Each Age Group (Age Group Total)	Proportion (%) in Each Age Group (95% CI)	Frequency in Each Age Group (Age Group Total)	Proportion (%) in Each Age Group (95% CI)
Men	20 to 34	1886 (11,638)	16.2 (15.5–16.9)	4737 (26,539)	17.8 (17.4–18.3)	100 (1886)	5.3 (4.3–6.3)	65 (100)	65.0 (55.7–74.3)
	35 to 49	6409 (18,057)	35.5 (34.8–36.2)	13,379 (37,055)	36.1 (35.6–36.6)	826 (6409)	12.9 (12.1–13.7)	213 (826)	25.8 (22.8–28.8)
	50 to 64	21,193 (33,253)	63.7 (63.2–64.2)	31,559 (52,918)	59.6 (59.2–60.0)	8236 (21,193)	38.9 (38.2–39.5)	1884 (8236)	22.9 (22.0–23.8)
	At least 65	4712 (6104)	77.2 (76.1–78.2)	5707 (8359)	68.3 (67.3–69.3)	2611 (4712)	55.4 (54.0–56.8)	741 (2611)	28.4 (26.7–30.1)
Women	20 to 34	670 (16,301)	4.1 (3.8–4.4)	1811 (36,669)	4.9 (4.7–5.2)	118 (670)	17.6 (14.7–20.5)	102 (118)	86.4 (80.3–92.6)
	35 to 49	5638 (27,642)	20.4 (19.9–20.9)	12,274 (54,311)	22.6 (22.2–23.0)	993 (5638)	17.6 (16.6–18.6)	331 (993)	33.3 (30.4–36.3)
	50 to 64	26,121 (45,586)	57.3 (56.8–57.8)	39,539 (72,387)	54.6 (54.3–55.0)	11,217 (26,121)	42.9 (42.3–43.5)	2836 (11,217)	25.3 (24.5–26.1)
	At least 65	7129 (8925)	79.9 (79.0–80.7)	8517 (12,191)	69.9 (69.0–70.7)	4418 (7129)	62.0 (60.8–63.1)	1205 (4418)	27.3 (26.0–28.6)

**Table 3:** The means and proportions of risk factors by sex for the survey years of 1975, 1990, 2000, and 2015.

Survey Year		1975		1990		2000		2010	
		Men	Women	Men	Women	Men	Women	Men	Women
Mean age in years (SD)		44.6 (15.2)	44.5 (15.3)	50.1 (15.8)	50.1 (15.9)	54.1 (16.2)	53.4 (15.9)	59.4 (16.0)	58.1 (16.0)
Mean salt in g/day (SD)		NA	NA	NA	NA	14.3 (6.0)	12.5 (5.0)	11.8 (4.6)	9.7 (3.8)
Mean BMI in kg/m^2^ (SD)		22.2 (2.9)	22.4 (3.3)	22.9 (3.0)	22.7 (3.3)	23.5 (3.2)	22.9 (3.5)	23.8 (3.2)	22.7 (3.5)
BMI frequency (%)	Underweight	680 (7.6%)	1224 (9.7%)	246 (6.2%)	439 (8.1%)	97 (4.0%)	290 (8.5%)	61 (3.6%)	224 (9.6%)
	Normal weight	6758 (76.0%)	8948 (70.7%)	2822 (70.9%)	3783 (69.7%)	1617 (66.7%)	2311 (67.5%)	1068 (63.8%)	1569 (67.5%)
	Obese	1456 (16.4%)	2476 (19.6%)	913 (22.9%)	1205 (22.2%)	711 (29.3%)	824 (24.1%)	546 (32.6%)	533 (22.9%)
Smoking frequency (%)	Non-smoker	NA	NA	1755 (44.0%)	4890 (90.0%)	1310 (54.0%)	3045 (89.0%)	440 (26.3%)	1948 (83.7%)
	Current smoker	NA	NA	2230 (56.0%)	542 (10.0%)	1115 (46.0%)	378 (11.0%)	1230 (73.7%)	379 (16.3%)
Drinking alcohol frequency (%)	Less than 4 times a week	NA	NA	1777 (44.6%)	5089 (93.7%)	1170 (48.3%)	3113 (91.0%)	483 (28.9%)	1513 (65.0%)
	More than 4 times a week	NA	NA	2208 (55.4%)	343 (6.3%)	1254 (51.7%)	309 (9.0%)	1187 (71.1%)	815 (35.0%)
Exercise frequency (%)	Less than 3 times a week	NA	NA	3063 (76.9%)	4414 (81.3%)	1626 (67.1%)	2454 (71.7%)	1063 (63.6%)	1638 (70.4%)
	More than 3 times a week	NA	NA	922 (23.1%)	1018 (18.7)	796 (32.9%)	967 (28.3%)	609 (36.4%)	690 (29.6%)
Occupation frequency (%)	Primary industry	1218 (14.9%)	1418 (12.4%)	425 (12.5%)	415 (8.5%)	184 (10.1%)	142 (4.8%)	145 (8.8%)	78 (3.4%)
	Secondary industry	2378 (29.1%)	1676 (14.7%)	951 (27.9%)	914 (18.8%)	457 (25.2%)	467 (15.8%)	231 (14.0%)	105 (4.5%)
	Tertiary industry	4572 (56.0%)	8312 (72.9%)	2028 (59.6%)	3532 (72.7%)	1176 (64.7%)	2344 (79.4%)	1277 (77.3%)	2137 (92.1%)

**Table 4:** The adjusted odds ratios for the prevalence of hypertension and the proportion of medicated and controlled hypertension for the survey years 1986 to 2010.

		Prevalence of Hypertension (*n* = 144,299)		Proportion of Medicated Hypertension (*n* = 109,229)		Proportion of Controlled Hypertension (*n* = 28,301)	
		OR (95%CI)	*p*-Value	OR (95%CI)	*p*-Value	OR (95%CI)	*p*-Value
Survey year	Per increase of one year	0.99 (0.99–0.99)	<0.001	1.02 (1.02–1.02)	<0.001	1.05 (1.04–1.05)	<0.001
Sex	Male	1.35 (1.31–1.39)		0.96 (0.92–1.00)	0.05	1.06 (0.97–1.15)	0.22
	Female	1	NA	1	NA	1	NA
Age (years)	20 to 34	1	NA	NA	NA	NA	NA
	35 to 49	3.22 (3.07 to 3.37)	<0.001	1	NA	1	NA
	50 to 64	13.49 (12.89–14.13)	<0.001	6.47 (6.13–6.83)	<0.001	0.56 (0.50–0.63)	<0.001
	at least 65	42.85 (39.93–46.00)	<0.001	19.09 (17.75–20.53)	<0.001	0.54 (0.47–0.62)	<0.001
BMI	Underweight	0.54 (0.51–0.57)	0.04	0.58 (0.53–0.64)	<0.001	1.32 (1.10–1.59)	0.003
	Normal	1	NA	1	NA	1	NA
	Obese	2.55 (2.48–2.62)	<0.001	2.07 (1.99–2.14)	<0.001	0.75 (0.70–0.81)	<0.001
Smoking	Non-smoker	1	NA	1	NA	1	NA
	Current smoker	0.92 (0.89–0.94)	<0.001	0.75 (0.72–0.79)	<0.001	0.97 (0.87–1.07)	0.49
Drinking alcohol	Less than 4 times a week	1	NA	1		1	NA
	At least 4 times a week	1.52 (1.47–1.57)	<0.001	1.21 (1.15–1.27)	<0.001	0.69 (0.63–0.77)	<0.001
Exercise	Less than 3 times a week	NA	NA	1	NA	1	NA
	At least 3 times a week	NA	NA	1.09 (1.04–1.13)	<0.001	1.09 (1.01–1.17)	0.03
Salt intake	Per 1 g/day	NA	NA	NA	NA	NA	NA
Occupation	Primary industry	1.14 (1.09–1.19)	<0.001	1.13 (1.07–1.19)	<0.001	0.99 (0.89–1.10)	0.83
	Secondary industry	1.00 (0.97–1.03)	0.99	0.83 (0.79–0.87)	<0.001	0.86 (0.77–0.96)	0.007
	Tertiary industry	1	NA	1	NA	1	NA
